# Overweight and obesity in children aged 3–13 years in urban Cameroon: a cross-sectional study of prevalence and association with socio-economic status

**DOI:** 10.1186/s40608-017-0146-4

**Published:** 2017-02-01

**Authors:** Simeon-Pierre Choukem, Josiane Kamdeu-Chedeu, Sam D. Leary, Yannick Mboue-Djieka, Daniel N. Nebongo, Christoph Akazong, Yacouba N. Mapoure, Julian P. Hamilton-Shield, Jean-François Gautier, Jean Claude Mbanya

**Affiliations:** 1 Health and Human Development (2HD) Research Group, Douala, Cameroon; 2 Department of Internal Medicine, Douala General Hospital, P.O. Box 4856, Douala, Cameroon; 3 Department of Internal Medicine and Pediatrics, Faculty of Health Sciences, University of Buea, Buea, Cameroon; 4 Faculty of Medicine and Pharmaceutical Sciences, University of Douala, Douala, Cameroon; 5 Biomedical Research Unit in Nutrition, Diet and Lifestyle, University of Bristol, Bristol, UK; 6 Department of Pediatrics, Douala General Hospital, Douala, Cameroon; 7 Department of Diabetes, Endocrinology and Nutrition, Assistance Publique - Hôpitaux de Paris, Lariboisière Hospital, University Paris-Diderot Paris-7, 2 rue Ambroise Paré, 75010 Paris, France; 8 National Centre of Obesity, Diabetes and Endocrinology, Yaoundé Central Hospital, Yaoundé, Cameroon; 9 Laboratory of molecular and metabolic medicine, Biotechnology Center, University of Yaoundé 1, Yaoundé, Cameroon; 10 Department of Internal Medicine and Subspecialties, Faculty of Medicine and Biomedical Sciences, University of Yaoundé 1, Yaoundé, Cameroon

**Keywords:** Overweight, Obesity, Children, Adolescent, Prevalence, Socioeconomic status, Cameroon

## Abstract

**Background:**

Childhood overweight/obesity is increasing rapidly in developing countries. There is a need to provide more evidence on its burden in sub-Saharan Africa, and to identify associated factors in order to set preventive measures. We aimed to determine the prevalence of overweight/obesity and assess its association with the socioeconomic status in nursery and primary school children in urban Cameroon.

**Methods:**

In this cross-sectional study, we included by multi-staged cluster random sampling 1343 children from high (HSES, *n* = 673) and low (LSES, *n* = 670) socioeconomic status schools in Douala. Parent/child demographic data were collected, and children’s anthropometric parameters were measured using validated methods. The World Health Organization body mass index-for-age reference curves were used.

**Results:**

The prevalence of overweight/obesity was 12.5% (13.2% in girls, 11.8% in boys). The risk of overweight/obesity was 2.40 (95% CI 1.70, 3.40) higher in HSES children compared to LSES after adjusting for age and gender. However this association was attenuated to 1.18 (95% CI 0.59, 2.35) once adjustment had been made for a range of potential confounders.

**Conclusions:**

Overweight/obesity is relatively common in sub-Saharan African children and prevalence is associated with HSES. However, this association may be mediated by sweet drink consumption, passive means of travel to school and not doing sport at school. We suggest that these potentially modifiable behaviors may be effective targets for obesity prevention. Further studies should specifically focus on unhealthy behaviors that mediate overweight/obesity as well as other non communicable diseases in children.

**Electronic supplementary material:**

The online version of this article (doi:10.1186/s40608-017-0146-4) contains supplementary material, which is available to authorized users.

## Background

The prevalence of overweight and obesity in children has dramatically increased over the past two decades [1]. In 2010, 43 million children were overweight or obese −35 million of whom lived in developing countries- and this number is expected to reach 60 million by 2020 [1]. Overweight or obese children are likely to remain so in adulthood and are also at high risk of developing non communicable diseases like diabetes, hypertension, cardiovascular diseases and cancers [2]. These non-communicable diseases are increasing alarmingly in sub-Saharan Africa (SSA) where infectious diseases and undernutrition in children still constitute the major focus of public health policies [3].

A recent systematic review showed rising trends of overweight and obesity in SSA children over time [4]. However, studies often had small sample size, lacked consistency regarding a uniform definition of overweight/obesity, or did not used standardized age groups; all the above make comparisons of studies inaccurate. Recent reports in Nigeria show prevalence of combined overweight and obesity of 3.5% in 1016 urban primary school children aged 6–10 years using the World Health Organization (WHO) reference [5] and 11.6% in 1302 urban primary school children 6–12 years using the CDC reference growth charts [6].

Socio-economic status (SES) is potentially a major risk factor for childhood obesity. In developed countries, recent studies confirm the well known inverse association, children from low socioeconomic status having a higher prevalence of obesity compared with those from higher socioeconomic status [7]. In low and middle income countries, data from South-Asian, the Far East and South-American populations demonstrated that high socio-economic status is associated with higher rates of overweight/obesity in children [8]. In low-income SSA countries studies are rare. A recent report in Cameroonian children aged 5 to 12 years showed findings similar to other low-income countries -high SES associated with overweight/obesity with an odds ratio (95% confidence interval) of 10.1 (5.4, 18.9) as compared with low SES. However, SES in that study was not a predefined predictor, and adjustment was made only for height standard deviations and birth weight [9]. Moreover, data are almost nonexistent for the age group under 5 years. As longitudinal studies have shown that early BMI or adiposity rebound by 43 months of age is one of the major independent risk factors for further childhood obesity, [10] this age group may be of particular interest for interventions and to estimate the future burden of obesity in older age groups.

Sustainable public health strategies aiming to tackle the emerging childhood and adolescent obesity in SSA can only be developed if sufficient evidence of its burden and risk factors is available. We therefore aimed to determine the prevalence of childhood overweight and obesity and assess its association with the socioeconomic status, adjusting for a wide range of confounders, in randomly selected nursery and primary schools in an urban area of Cameroon. We hypothesized that high SES is associated with overweight/obesity after adjustment for these confounders.

## Methods

### Study design and population

This was a cross-sectional study conducted in Douala, the biggest city of Cameroon with an estimated population of 2.446.945 inhabitants in 2011. Neighborhoods in Douala are classified by the National Institute of Statistics (NIS) as low, medium and high socio-economic status using various indicators [11]. The survey that provided data for this ranking was based on a random sampling of 7500 households per city (Douala and Yaoundé). The main indicators used in classification were the annual income and the housing status. The same NIS-based ranking of neighborhoods was used to select schools in a previous study that assessed the role of SES in adolescents’ nutritional status in Yaoundé [12]. The Primary school net enrolment ratio in Cameroon was estimated at 93.5% in 2008–2011 by the United Nations International Children’s Emergency Fund [13]. Thus, a sample from nursery and primary schools is likely to be representative of children living in Douala.

We performed a multi-staged cluster random sampling that consisted of: 1) random selection by balloting of one area of low socio-economic status (LSES) and one area of medium-to-high socio economic status (HSES) in the city of Douala; 2) census of all nursery and primary schools in each area and census of the number of pupils per school; 3) random selection of schools per area until the target sample size with an additional 25% was reached. Five schools were selected in *Bonamoussadi-Makepe* (HSES) and five in *Boko Village* (LSES). Because *Bonamoussadi-Makepe* is a mixture of middle and high SES, we merged the two SES in one group. All children aged 3 years and above were invited to participate through a letter to their parents.

### Sample size and power calculation

According to the EDS-MICS (*Enquête Démographique et de Santé et à Indicateurs Multiples*) survey in 2011, [14] the prevalence of childhood overweight and obesity in Cameroon was 6%. Our study was designed to detect a 40% higher prevalence in children from HSES schools (7%) compared with those from LSES schools (5%), with a type I error of 5%, two-sided tests, and a power of 80%. The minimum sample was 619 participants per group (total 1238).

### Data collection and classification of the nutritional status

Data were collected between February and March 2013 using a questionnaire. Information letters, consent forms (in duplicates) and questionnaires were handed to each class teacher who put them in each child’s school bag along with their daily homework. Parents had to retrieve these documents and read and sign the information letter. Those who agreed to let their children participate in the study also signed the informed consent forms, filled in the first part of the questionnaire and put them in their child’s school bag. These forms were collected back by the teacher and handed to our team. A copy of the signed informed consent was returned to parents through the same means. The questionnaire contained personal and social data on the child (date of birth, birth weight, eating habits including early feeding, physical activity during school and leisure time, means of travel to school, sleeping habits, electronic and television use habits, and receipt of pocket money), their mother (age, education level, smoking and alcohol habits, weight and height), and their father (education level). Physical activity in school was confirmed by the teacher.

Children thereafter had their anthropometry (weight in kg and standing height in cm) measured within 7 days by a team of trained doctors who followed pre-established standard operating procedures. The weight to the nearest 100 g was measured using a Camry® bathroom scale, and the height to the nearest 0.5cmwas measured using a Leicester® stadiometer. The body mass index (BMI) was calculated (kg/m^2^). All measurements were made once with the child bare foot and wearing light clothes. All children were measured using the same brand of scale and stadiometer. The scales were readjusted to zero after each measurement.

To determine the participant’s nutritional status, the BMI was plotted against the WHO body mass index-for-age reference curves for 0–5 years[15] and 5–19 years [16].

### Statistical analysis

Stata version 13 was used for data analysis. Continuous variables (all approximately normally distributed) were summarized by means and standard deviations, and categorical variables were summarized by proportions. Variables were summarized for the whole group, and also separately for HSES and LSES; the latter were compared using t-tests or chi-square tests as appropriate. The prevalence of overweight/obesity was calculated using proportions with 95% confidence intervals (CI); values are given for the whole cohort, boys and girls separately, each age group separately, and each social class group separately. Associations between each variable and overweight/obesity were assessed using random effects logistic models; random effects were used to account for the dependence of children within the same school. A series of models were fitted with socio-economic status as the predictor and overweight/obesity as the outcome, adjusting for the following potential confounders: model 1 adjusted only for age and gender; model 2 adjusted for age, gender and early life factors (birth weight, type of feeding from 0 to 6 months); model 3 adjusted for age, gender and parental factors (mother’s age, BMI, education level, alcohol consumption, and father’s education level); model 4 adjusted for age, gender, early life factors and parental factors; model 5 adjusted for age, gender and current child factors (number of meals per day, fruit consumption, sweet drink consumption, physical activity at school and leisure time, travel means to school, sleeping habits, electronic and television use habits, time child wakes up, daytime sleep and receipt of pocket money); and model 6 adjusted for all potential confounders. Variance inflation factors were calculated for each potential confounder, and as the maximum value was 1.6, all variables were included in the final model.

### Ethical considerations

Ethical approval was obtained from the National Ethics Committee (N° 2013/05/322/CNERSH/SP) and administrative clearances from the regional delegation for basic education and directors of selected schools. Children were included only if parents signed the information letter and the informed consent form.

## Results

### General characteristics of participants

**Fig. 1:**
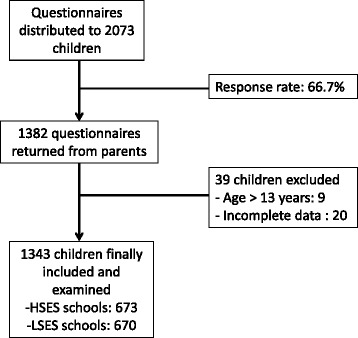
Inclusion procedure of children in the study. HSES: high socioeconomic status; LSES: low socioeconomic status

We included 1343 children aged 3–13 years −673 from middle-to-high socioeconomic status (HSES) schools and 670 from low socioeconomic level (LSES) schools (Fig. 1). Compared with LSES children, children from HSES were less likely to have been breast-fed during the first six months of life, more likely to have three regular meals per day, to have regular fruit consumption and to consume sweet drinks regularly. They also were more likely to have a daytime ‘nap’, have greater ‘electronic screen-time’, and to be passively transported to school, and less likely to play sport at school and to have pocket money (Additional file 1). Parents of HSES children had higher level of education and their mothers drank less alcohol (Additional file 1). Children from HSES schools had higher weight, height and BMI compared with those from LSES schools (Table 1). The distribution of other categorical and continuous variables was similar between the two groups (Additional file 1 and Table 1).

**Table 1:** Summary of all continuous variables for the whole cohort and separately for HSES and LSES

	Whole cohort		HSES		LSES		*p* value*
	*N*	Mean (SD)	*N*	Mean (SD)	*N*	Mean (SD)	
Age (years)	1343	8.0 (2.4)	673	8.0 (2.3)	670	8.1 (2.5)	0.4
Birth weight (kg)	960	3.3 (0.6)	482	3.2 (0.6)	478	3.3 (0.6)	0.2
Maternal age (years)	1131	35.0 (6.0)	610	35.1 (6.1)	521	35.0 (5.9)	0.8
Maternal weight (kg)	1081	72.1 (12.1)	556	72.5 (12.4)	482	71.6 (11.9)	0.2
Maternal height (cm)	1038	163.1 (8.2)	581	163.5 (8.5)	500	162.6 (7.9)	0.1
Maternal BMI (kg/m^2^)	993	27.3 (5.0)	539	27.3 (5.1)	454	27.3 (4.9)	0.99
Child weight (kg)	1343	25.3 (7.9)	673	26.4 (8.4)	670	24.2 (7.2)	<0.001
Child height (cm)	1343	124.6 (14.6)	673	126.2 (14.7)	670	123.0 (14.3)	<0.001
Child BMI (kg/m^2^)	1343	15.9 (2.2)	673	16.2 (2.4)	670	15.7 (1.9)	<0.001
BMI children < 6 years	222	15.5 (2.0)	104	15.7 (2.2)	118	15.4 (1.9)	0.35
BMI children 6–10 years	896	15.7 (2.1)	466	16.0 (2.3)	430	15.5 (1.7)	<0.001
BMI children >10 years	225	17.1 (2.4)	103	17.7 (2.6)	122	16.7 (2.1)	0.002

*BMI* body mass index, *HSES* High socioeconomic status, *LSES* Low socioeconomic status, *SD* standard deviation; *p-value from t-tests, HSES vs. LSES

### Prevalence of overweight and obesity

The overall prevalence of overweight (9.6%) and obesity (2.9%) combined was 12.5% (95% CI 10.8, 14.4) (Table 2). This prevalence was 11.8% in boys and 13.2% in girls, 17.1% in children from HSES schools and 7.9% in those from LSES schools (Table 2).

**Table 2:** Prevalence of overweight/obesity: whole cohort, by gender, by age-group and by SES

	Prevalence (95% CI)		
Whole cohort			
Overweight/obesity	12.5 (10.8, 14.4)%		
Normal/underweight	87.5 (85.6, 89.2)%		
Obesity	2.9 (2.1, 4.0)%		
Overweight	9.6 (8.1, 11.3)%		
Normal weight	83.6 (81.5, 85.5)%		
Underweight	3.9 (3.0, 5.0)%		
By gender	Boys	Girls	
Overweight/obesity	11.8 (9.5, 14.5)%	13.2 (10.9, 16.0)%	
Normal/underweight	88.2 (85.5, 90.5)%	86.8 (84.0, 89.1)%	
Obesity	2.6 (1.6, 4.1)%	3.2 (2.1, 4.8)%	
Overweight	9.2 (7.2, 11.6)%	10.0 (8.0, 12.5)%	
Normal weight	84.4 (81.4, 87.0)%	82.8 (79.8, 85.5)%	
Underweight	3.8 (2.6, 5.6)%	3.9 (2.7, 5.7)%	
By age groups	<6 years	6–10years	>10 years
Overweight/obesity	17.6 (13.1, 23.2)%	11.6 (9.7, 13.9)%	11.1 (7.6, 16.0)%
Normal/underweight	82.4 (76.8, 86.9)%	88.4 (86.1, 90.3)%	88.9 (84.0, 92.4)%
Obesity	4.1 (2.1, 7.6)%	2.9 (2.0, 4.2)%	1.8 (0.7, 4.7)%
Overweight	13.5 (9.6, 18.7)%	8.7 (7.0, 10.7)%	9.3 (6.1, 13.9)%
Normal weight	78.8 (72.9, 83.7)%	84.8 (82.3, 87.0)%	83.6 (78.1, 87.8)%
Underweight	3.6 (1.8, 7.1)%	3.6 (2.5, 5.0)%	5.3 (3.0, 9.2)%
By SES	HSES	LSES	
Overweight/obesity	17.1 (14.4, 20.1)%	7.9 (6.1, 10.2)%	
Normal/underweight	82.9 (79.9, 85.6)%	92.1 (89.8, 93.9)%	
Obesity	4.3 (3.0, 6.1)%	1.5 (0.8, 2.8)%	
Overweight	12.8 (10.5, 15.5)%	6.4 (4.8, 8.5)%	
Normal weight	78.8 (75.5, 81.7)%	88.5 (85.9, 90.7)%	
Underweight	4.2 (2.9, 6.0)%	3.6 (2.4, 5.3)%	

*CI* confidence interval, *HSES* High socioeconomic status, *LSES* Low socioeconomic status, *SES* Socioeconomic status

### Associations between potential confounders and overweight/obesity

Based on univariate models (Table 3), lower age, higher birth weight, higher sweet drink consumption, passive means of travel to school, not doing sport at school and higher maternal BMI were positively associated with overweight and obesity. There were no strong associations between any of the other potential confounders and overweight/obesity.

**Table 3:** Associations between potential confounders and overweight/obesity in univariate models

Potential confounders	Categories	OR (95% CI)	*P* value*
Gender (vs. male)	Female	1.16 (83.9, 1.62)	0.4
Age (per year)	-	0.92 (0.86, 0.98)	0.01
Birthweight (per 100 g)	-	1.01 (1.002, 1.009)	0.001
Type of feeding from 0 to 6 months (vs. breast milk)	Formula	1.92 (0.79, 4.68)	0.2
	Breast milk and formula	1.15 (0.81, 1.63)	0.4
Number of meals per day (vs. 1–2)	3	1.22 (0.72, 2.10)	0.06
	4+	1.76 (0.94, 3.30)	
Fruit consumption (vs. 4–7 times/week)	1-3 times/week	0.54 (0.35, 0.85)	0.1
	<1/week	0.63 (0.40, 0.99)	
Sweet drink consumption (vs. never/rarely)	Often	1.42 (0.997, 2.02)	0.01
	Everyday	1.98 (1.12, 3.51)	
Leisure time sport (vs. yes)	No	0.97 (0.69, 1.35)	0.8
Passive travel to school (vs. walk/cycle)	Motorcycle/taxi/car/public transport	1.95 (1.28, 2.95)	0.002
School sport (vs. yes)	No	2.08 (1.24, 3.48)	0.01
Time watching screen (vs. <1 h/day)	1-2 h/day	1.16 (0.77, 1.74)	0.2
	2+ hour/day	1.35 (0.87, 2.11)	
Time child wakes up (vs. between 6 am and 7 am)	Between 3 am and 5.55 am	0.82 (0.57, 1.18)	0.3
Daytime sleep (vs. yes)	No	0.94 (0.66, 1.35)	0.7
Pocket money (vs. yes)	No	1.02 (0.72, 1.44)	0.9
Maternal age (per year)	-	1.01 (0.99, 1.04)	0.3
Maternal BMI (per kg/m^2^)	-	1.06 (1.02, 1.09)	0.002
Maternal education level (vs. university)	High school	1.09 (0.67, 1.78)	0.1
	Secondary school	0.82 (0.50, 1.36)	
	None/Primary school	0.68 (0.37, 1.25)	
Maternal alcohol consumption (vs. yes)	No	1.50 (0.92, 2.43)	0.1
Paternal education level (vs. university)	High school Secondary	0.78 (0.52, 1.18)	0.08
	school	0.59 (0.36, 0.97)	
	None/Primary school	0.75 (0.41, 1.37)	

*OR* odds ratio, *CI* confidence interval; *p-value for trend if ordinal variable has more than two categories

### Associations between socio-economic status and overweight/obesity

After adjustment for only age and gender (model 1), HSES was associated with a 2.40 (95%CI 1.70, 3.40) higher risk of overweight/obesity compared to LSES (Table 4). After additional adjustment for either early life factors, parental factors, or current child factors, the risk attenuated a little, to approximately twice as high for HSES compared to LSES (Table 4). However, after adjustment for all potential confounders the risk attenuated to only 1.18 (95% CI 0.59, 2.35) times higher for HSES compared to LSES (Table 4). Adjusting for all confounders reduced the sample size from 1343 to 620, however when model 1 was re-fitted restricted to this sample, the risk of overweight/obesity was still almost doubled (OR = 1.95; 95%CI 1.18, 3.23; *p* = 0.01) for HSES compared to LSES.

**Table 4:** Associations between SES and overweight/obesity in multivariate models

Outcome HSES vs. LSES	Adjusted OR	95% CI	*P* value
Model 1 [Adjusted for age and gender]	2.40	1.70, 3.40	<0.001
Model 2 [Adjusted for age, gender and early life factors*]	2.23	1.45, 3.45	<0.001
Model 3 [Adjusted for age, gender and parental factors^a^]	2.20	1.38, 3.52	0.001
Model 4 [Adjusted for age, gender, early life factors and parental factors]	1.94	1.13, 3.36	0.02
Model 5 [Adjusted for age, gender and current child factors^a^]	1.93	1.21, 3.07	0.005
Model 6 [Adjusted for all potential confounders]	1.18	0.59, 2.35	0.6

*OR* odds ratio, *CI* confidence interval, *SES* socioeconomic status

*Type of feeding 0–6 months, birth weight

^†^Maternal age, maternal BMI, maternal education, maternal alcohol consumption, paternal education

^a^Number of meals per day, fruit consumption, sweet drink consumption, leisure time sport, travel to school, school sport, time watching screen, time child wakes up, daytime nap, pocket money

## Discussion

We have shown in this study that the prevalence of overweight and obesity in nursery and primary school children aged 3–13 years was 12.5%, without any statistical evidence of a gender difference. This prevalence is twice that reported in the EDS-MICS; the EDS-MICS included rural areas of the country, which diluted the prevalence [14]. We have also shown that high socio-economic status (HSES) was strongly associated with overweight/obesity, with HSES schools children being almost two and half times more likely to be overweight or obese than those from low socio-economic status (LSES) schools. However, the increased risk with the HSES appeared to be driven by other factors, since the association was attenuated when all potential confounders were included in the regression model.

The prevalence we report here indicates a high burden of overweight and obesity in children in this urban area of sub-Saharan Africa (SSA) where infectious diseases and malnutrition are yet to be tackled [3]. Another recent study in Cameroon found a prevalence of overweight and obesity of 17.9% in urban children aged 8–15 years using the WHO references [17]. Studies in other sub-Saharan African countries have reported prevalence levels of overweight and obesity that vary according to the area and criteria used to define it: 3.5% in 1016 primary school children aged 6–10 years in urban South-western Nigeria using the WHO references; [5] 11.6% in 1302 primary school children aged 6–12 years in urban south-eastern Nigeria using the CDC references, [6] and 13.4% in the subgroup of 4833 Black children aged 6–13 years in South Africa, using the IOTF criteria [18]. All the aforementioned studies used objective measurements to define overweight and obesity. The prevalence in our population is therefore close to the upper limit of the range in SSA. Figures are much more alarming in developed countries. In the USA for instance, overweight and obesity concerned 26.7% of children aged 2–5 years and 32.6% of those aged 6–11 years in 2009–2010 [19].

Studies that have included children below 5 years of age in SSA are very rare, but show prevalence of overweight and obesity as high as 22% [20]. In that specific age group, we also observed a higher prevalence of overweight/obesity (17.6%) compared with older children (11.6% in 6–10 year-old children and 11.1% in 10–13 year-old ones). This may indicate a future worsening of overweight/obesity in older age groups as shown in the Avon Longitudinal Study of Parents and Children cohort study where early BMI or adiposity rebound by 43 months of age was one of the major independent risk factors (15-fold increased risk) for further childhood obesity [10].

We found that the socio-economic status (SES) was positively associated with overweight/obesity. Socio-economic status is considered as one of the major risk factors of childhood obesity. In developed countries, a negative gradient has long been described between the SES and overweight/obesity, and has recently been confirmed in children [7]. The relationship between the SES and overweight/obesity is known to be positive in adults in developing countries [21]. A similar pattern was observed recently in children aged 5–12 years in Cameroon, but SES was not a predefined predictor, and adjustment was made only for height standard deviations and birth weight [9]. Thus, whether the consideration of potential confounders would keep the independence of the association of childhood overweight/obesity with SES was not known. SES is a combination or a trigger of behavioral or parental factors that may individually play a direct role in the risk of overweight/obesity. Our observation of the attenuation of the association between SES and overweight/obesity in the model including all confounders suggest that other factors probably come into play. This attenuation was more prominent when current child factors were added to age and gender in the model, and there was no statistical evidence for an association between SES and overweight/obesity once all confounders were considered. It is possible that models 5 and 6 could be over-adjustments, and we hypothesize that current child factors, especially those that were strongly associated with overweight/obesity in the univariate model –higher sweet drink consumption, passive means of travel to school and not doing sport at school- are potential mediators in the association between HSES and overweight/obesity in our population. These factors are indeed indicators of HSES and have been reported by others to be associated with childhood overweight/obesity. For instance, consumption of sugary drinks was found to be strongly associated with childhood obesity in a systematic review of thirty studies [22]. Active transport to school is an important means to ensure permanent moderate physical activity in children [23]. Its role may be more prominent in a developing country like Cameroon where children in rural as well as urban areas may walk for miles every day to and from their financially accessible school. This finding also has public health relevance regarding the promotion of active transport by walking or cycling.

The major limitation of our study is its cross-sectional design that does not allow us to evaluate the impact of the SES and other potential risk factors we have identified. However, we have used a multistage cluster sampling to select a population that probably represent the general children population of the city, as the primary school net enrolment ratio in Cameroon was estimated at 93.5% in 2008–2011 [13]. Also, as parental data, birth weight and child demographic data were reported by parents, self-reporting bias is another potential limitation. To overcome this, we cross-checked the data with children’s school files in case of doubt. Amongst other potential limitations is the use of neighborhood as the main criterion for SES, as children from one neighborhood may go to school in another part of the city of different SES. In addition, the psychometric properties of the questionnaire have not been assessed independently in other studies. Finally, our sample may not be nationally representative so generalizability is currently unproven.

## Conclusions

Overweight/obesity is highly prevalent in urban sub-Saharan African children. It is strongly positively associated with SES, but this association may be mediated by behavioral socioeconomic indicators like sweet drink consumption, passive mean of travel to school and not doing sport at school. Our results suggest that strategies to tackle children overweight/obesity in SSA should consider these modifiable factors. Further studies should specifically focus on unhealthy behaviors that mediate overweight/obesity as well as other non communicable diseases in children.

## Additional file

Additional file 1. Summary of all categorical variables; whole cohort and separately for HSES and LSES. HSES = High socioeconomic status; LSES = Low socioeconomic status; IQR = inter-quartile range; *p-value from chi-squared tests, HSES vs.LSES; ^†^of those who receive pocket money, median (IQR) = 150 (100, 200) FCFA. (DOCX 16 kb)

## Abbreviations

BMI: Body mass index
CDC: Centers for disease control and prevention
CI: Confidence interval
EDS-MICS: Enquête démographique et de santé et à indicateurs multiples
HSES: High socio-economic status
IOTF: International obesity task force
LSES: Low socio-economic status
OR: Odd ratio
SES: Socio-economic status
SSA: Sub-Saharan Africa
USA: United States of America
WHO: World health organization
