# Supplementary material for: Overweight and obesity in children aged 3–13 years in urban Cameroon: a cross-sectional study of prevalence and association with socio-economic status
Source: BMC Obes. 2017 Feb 1;4:7. doi: 10.1186/s40608-017-0146-4 (PMC5286775; doi:10.1186/s40608-017-0146-4)
Supplement: Additional file 1. — Summary of all categorical variables; whole cohort and separately for HSES and LSES. HSES = High socioeconomic status; LSES = Low socioeconomic status; IQR = inter-quartile range; *p-value from chi-squared tests, HSES vs.LSES; †of those who receive pocket money, median (IQR) = 150 (100, 200) FCFA. (DOCX 16 kb) [file 40608_2017_146_MOESM1_ESM.docx]

**Table 1**: Summary of all categorical variables; whole cohort and separately for HSES and LSES

|  | **Categories** | **Whole cohort** | | **HSES** | | **LSES** | | **P value*** |
| --- | --- | --- | --- | --- | --- | --- | --- | --- |
|  |  | **N** | **%** | **N** | **%** | **N** | **%** |  |
| **Gender** | Male  Female | 655  688 | 48.8  51.2 | 334  339 | 49.6  50.4 | 321  349 | 47.9  52.1 | 0.5 |
| **Age group** | < 5 years  6-10 years  >10 years | 222  896  225 | 16.5  66.7  16.6 | 104  466  103 | 15.5  69.2  15.3 | 118  430  122 | 17.6  64.2  18.2 | 0.1 |
| **Type of feeding from 0 to 6 months** | Breast milk  Formula  Breast milk and formula | 707  32  576 | 53.8  2.4  43.8 | 282  23  355 | 42.7  3.5  53.8 | 425  9  221 | 64.9  1.4  33.7 | <0.001 |
| **Number of meals per day** | 1 -2  3  4+ | 185  773  347 | 14.2  59.2  26.6 | 76  462  120 | 11.6  70.2  18.2 | 109  311  227 | 16.9  48.1  35.1 | <0.001 |
| **Fruit consumption** | 4-7 times/week  1-3 times/week  <1/week | 202  591  510 | 15.5  45.4  39.1 | 139  291  229 | 21.1  44.2  34.8 | 63  300  281 | 9.8  46.6  43.6 | <0.001 |
| **Sweet drink consumption** | Never/rarely  Often  Everyday | 614  599  100 | 46.8  45.6  7.6 | 288  301  72 | 43.6  45.5  10.9 | 326  298  28 | 50.0  45.7  4.3 | <0.001 |
| **Leisure time sport** | Yes  No | 625  678 | 48.0  52.0 | 320  339 | 48.6  51.4 | 305  339 | 47.4  52.6 | 0.7 |
| **Active travel to school** | Walk/cycle  Motorcycle/taxi/car/public transport | 960  352 | 73.2  26.8 | 339  319 | 51.5  48.5 | 621  33 | 95.0  5.1 | <0.001 |
| **School sport** | Yes  No | 1213  130 | 90.3  9.7 | 544  129 | 80.8  19.2 | 669  1 | 99.9  0.2 | <0.001 |
| **Time watching screen** | <1 hour/day  1-2 hour/day  2+ hour/day | 424  544  324 | 32.8  42.1  25.1 | 186  279  183 | 28.7  43.1  28.2 | 238  265  141 | 37.0  41.2  21.9 | 0.002 |
| **Time child wakes up** | Between 6am and 7am  Between 3am and 5.55am | 834  466 | 64.2  35.9 | 436  219 | 66.6  33.4 | 398  247 | 61.7  38.3 | 0.07 |
| **Daytime nap** | Yes  No | 836  434 | 65.8  34.2 | 441  197 | 69.1  30.9 | 395  237 | 62.5  37.5 | 0.01 |
| **Pocket money^†^** | Yes  No | 644  668 | 49.1  50.9 | 246  418 | 37.1  63.0 | 398  250 | 61.4  38.6 | <0.001 |
| **Maternal education highest level** | University  High school  Secondary school  None /Primary School | 207  352  458  251 | 16.3  27.8  36.1  19.8 | 160  215  201  73 | 24.7  33.1  31.0  11.3 | 47  137  257  178 | 7.6  22.1  41.5  28.8 | <0.001 |
| **Maternal smoking** | Yes  No | 10  1289 | 0.8  99.2 | 4  655 | 0.6  99.4 | 6  634 | 0.9  99.1 | 0.5 |
| **Maternal alcohol consumption** | Yes  No | 270  1011 | 21.1  78.9 | 91  559 | 14.0  86.0 | 179  452 | 28.4  71.6 | <0.001 |
| **Paternal education highest level** | University  High school  Secondary school  None/Primary school | 384  400  296  158 | 31.0  32.3  23.9  12.8 | 274  207  119  35 | 43.2  32.6  18.7  5.5 | 110  193  177  123 | 18.2  32.0  29.4  20.4 | <0.001 |

HSES = High socioeconomic status; LSES = Low socioeconomic status; IQR = inter-quartile range; *p-value from chi-squared tests, HSES vs.LSES; ^†^of those who receive pocket money, median (IQR) = 150 (100, 200) FCFA.
